# Association of Serum Gamma-Glutamyltransferase with In-hospital Heart Failure in Patients with ST-segment Elevation Myocardial Infarction Undergoing Primary Percutaneous Coronary Intervention

**DOI:** 10.31083/RCM25005

**Published:** 2025-01-08

**Authors:** An-Cheng Hou, Jian-Tong Hou, Wei-Ning Zhou, Yan-Jin Wei, Zhi-Hong Ou, Cun-Fei Liu

**Affiliations:** ^1^Department of Cardiology, Linyi People’s Hospital, Shandong Second Medical University, 276000 Linyi, Shandong, China; ^2^Department of Pathology, Linyi People’s Hospital, Shandong Second Medical University, 276000 Linyi, Shandong, China

**Keywords:** gamma-glutamyltransferase, ST-segment elevation myocardial infarction, percutaneous coronary intervention, heart failure

## Abstract

**Background:**

To explore the association between gamma-glutamyltransferase (GGT) and in-hospital heart failure (HF) in patients with ST-segment elevation myocardial infarction (STEMI) undergoing primary percutaneous coronary intervention (PCI).

**Methods:**

A total of 412 patients diagnosed with STEMI and treated with primary PCI were included in our study. Univariate and multivariate logistic regression models were used to evaluate the association between GGT and the risk of in-hospital HF in STEMI patients. The receiver operating characteristic (ROC) curve was used to assess the accuracy of GGT in predicting in-hospital HF.

**Results:**

The incidence of HF after STEMI increased significantly with increasing GGT tertiles (the first, second, and third tertile groups were 7.97%, 14.49%, and 18.38%, respectively; *p* = 0.039). Multivariate logistic regression analysis revealed that the risk of HF in the second and third GGT tertile groups was 2.51 times greater (95% CI, 1.06–5.96) and 2.77 times greater (95% CI, 1.13–6.81), respectively, than that in the first GGT tertile group. Each 1-unit increase in the lnGGT level was related to a 1.88-fold increased risk of HF (odds ratio, OR, 1.88; 95% CI, 1.19–2.96; *p* = 0.007). Restricted cubic splines suggested a linear relationship between GGT and in-hospital HF (*p* for nonlinearity = 0.158). The area under the curve was 0.607 (95% CI, 0.558–0.654; *p* = 0.007) when GGT was used to predict in-hospital HF, with a sensitivity of 57.14% and a specificity of 64.04%. Moreover, the incidence of HF significantly increased in-hospital death risk (OR, 7.75; 95% CI, 1.87–32.12; *p* = 0.005).

**Conclusions:**

GGT is positively associated with in-hospital HF and is an independent risk factor for in-hospital HF in STEMI patients.

## 1. Introduction

Myocardial infarction remains one of the most common causes of heart failure (HF) worldwide. The mortality rate of acute myocardial infarction has decreased significantly with the development of pharmacological and nonpharmacological treatments such as percutaneous coronary intervention (PCI) [1]. However, the incidence of HF after myocardial infarction remains high, and the occurrence of HF after myocardial infarction significantly increases the risk of short-term and long-term mortality in these patients [2, 3].

In addition to being a biomarker of liver disease and alcohol consumption, gamma-glutamyltransferase (GGT) is closely related to cardiovascular diseases, such as coronary heart disease, atrial fibrillation, and HF [4, 5, 6]. For example, Jeon *et al*. [4] reported that elevated GGT levels might be useful predictors for the development of atrial fibrillation. Persistent exposure to high GGT levels was associated with increased risks of myocardial infarction, stroke, cardiovascular diseases, and death [7]. Higher GGT levels are also related to future risks of HF [8].

However, the relationship between GGT and HF and complications toward myocardial infarction is unclear, particularly in patients with ST-segment elevation myocardial infarction (STEMI) undergoing primary PCI. Therefore, we explored the relationship between GGT and in-hospital HF in STEMI patients undergoing primary PCI.

## 2. Methods

### 2.1 Study Populations

All the subjects were diagnosed with STEMI between January 2019 and December 2020 in the cardiac intensive care unit of the Linyi People’s Hospital. STEMI was defined based on the fourth universal definition of myocardial infarction [9]. The enrolled patients received standard PCI procedures by experienced surgeons according to clinical practice guidelines [10]. Only culprit vessels in STEMI patients were affected, and the thrombolysis in myocardial infarction (TIMI) flow grade of these subjects was restored to TIMI flow grade 3 after PCI surgery. People with the following previous history were excluded: A known history of prior myocardial infarction, heart failure, active infections, or malignancy. Finally, 412 STEMI patients were included in our study (the flowchart is shown in **Supplementary Fig. 1**). The Ethics Committee of the Linyi People’s Hospital (No YX200312) approved this study. The data are anonymized, meaning written informed consent was waived.

The main endpoint was new-onset HF at the time of admission or during hospitalization after primary PCI, based on the Killip classification [11] (class I: no HF signs; class II: emergence with third heart sound (S3) or lung rales; class III: acute pulmonary edema; class IV: manifestations of cardiogenic shock). People with Killip classifications II–IV were considered to present with in-hospital HF.

### 2.2 Data Collection and Laboratory Analyses

Experienced interventional experts performed the primary PCI procedures for STEMI patients. Surgical information was obtained from the medical records. Venous blood samples were obtained for laboratory analyses of hematologic parameters, liver and kidney function biomarkers, troponin-T, N-terminal pro-B-type natriuretic peptide (NT-proBNP), and blood lipids. All patients received transthoracic Doppler echocardiography during the first 24 hours after admission. In addition, baseline clinical information (history of hypertension, diabetes, dyslipidemia, smoking, etc.) was also collected for each patient.

### 2.3 Statistical Analysis

Continuous variables are presented as the mean (SD) or median (IQR), and categorical variables are reported as numbers and percentages. The Chi-square test was chosen for comparisons of categorical variables. Student’s *t*-test or the Mann‒Whitney test was used for normally distributed continuous variables or skewed data between two groups, respectively. One-wayanalysis of variance (ANOVA), or the Kruskal‒Wallis test, was used to compare multiple groups. Univariate and multiple logistic regression analyses evaluated the association between GGT levels and in-hospital HF risk. Age and sex were adjusted for in multivariate analysis Model 2. Model 3 was adjusted for the following covariates: Age, sex, hypertension, diabetes, anterior myocardial infarction, and alanine aminotransferase. In addition, the receiver operating characteristic (ROC) curve was chosen to evaluate the sensitivity and specificity of the NLR and the optimal cut-off value for the prediction of in-hospital HF. Potential nonlinear associations between GGT levels and in-hospital HF risk were evaluated via restricted cubic splines (RCSs) with three nodes (10th, 50th, and 90th percentiles).

A *p*-value < 0.05 was considered statistically significant. Statistical analyses were conducted using SPSS software 18.0 (SPSS Inc., Chicago, IL, USA) and the statistical package R 4.2 (R Foundation for Statistical Computing, Vienna, Austria).

## 3. Results

### 3.1 Baseline Characteristics of the Enrolled Subjects

The baseline characteristics of the enrolled subjects are shown in Table 1. HF occurred in 56 subjects, and the prevalence of HF was 13.59% among all STEMI patients. Compared with those in the non-HF group, the average age of patients in the HF group was greater (65.13 years *vs*. 58.12 years), with a greater proportion of women (44.64% *vs*. 17.42%), hypertension patients (67.86% *vs*. 41.29%), diabetes mellitus patients (39.29% *vs*. 19.10%), anterior myocardial infarction patients (76.79% *vs*. 51.69%), and death patients (8.93% *vs*. 1.12%). Furthermore, compared with those in the non-HF group, the left ventricular ejection fraction (LVEF) in the HF group was significantly lower (47.00% *vs*. 53.10%, *p* < 0.001). In addition, people in the HF group had higher levels of C-reactive protein (CRP), fasting blood glucose (FBG), NT-proBNP, alanine aminotransferase (ALT), and creatinine (*p* < 0.01 or 0.05). The two groups had no significant differences in diastolic blood pressure, systolic blood pressure, troponin T, or blood lipids (*p* > 0.05).

**Table 1.** **Characteristics of the study subjects**.

	HF (n = 56)	Non-HF (n = 356)	*p*-value
Female (%)	25 (44.64%)	62 (17.42%)	<0.001
Age (year)	65.30 ± 10.94	58.12 ± 12.80	<0.001
Diabetes mellitus (%)	22 (39.29%)	68 (19.10%)	<0.001
Hypertension (%)	38 (67.86%)	147 (41.29%)	<0.001
SBP (mmHg)	121.36 ± 19.64	124.72 ± 18.27	0.206
DBP (mmHg)	76.52 ± 11.20	79.85 ± 13.31	0.076
Anterior myocardial infarction (%)	43 (76.79)	184 (51.69)	<0.001
Death during hospitalization (%)	5 (8.93%)	4 (1.12%)	<0.001
LVEF (%)	47.00 ± 7.73	53.10 ± 5.71	<0.001
CRP (mg/L)	16.69 (5.23–39.85)	4.31 (3.10–9.69)	<0.001
NT-pro BNP (pg/mL)	2841 (1269–5624)	780 (260–1537)	<0.001
TnT (ng/mL)	4.12 ± 0.84	4.05 ± 0.76	0.473
WBCs (×10^9^/L)	13.24 ± 4.86	10.08 ± 3.05	<0.001
ALT (IU/L)	55.95 (34.25–86.5)	36.70 (25.85–56.38)	<0.001
GGT (IU/L)	28.10 (20.00–37.95)	21.65 (16.00–32.78)	0.01
FBG (mmol/L)	10.23 ± 3.99	7.27 ± 2.89	<0.001
Cr (µmol/L)	76.42 ± 28.40	66.64 ± 17.04	0.015
TG (mmol/L)	1.40 ± 0.65	1.72 ± 1.49	0.007
TC (mmol/L)	4.57 ± 1.18	4.69 ± 1.18	0.504
LDL-C (mmol/L)	3.08 ± 1.10	3.07 ± 0.89	0.925
HDL-C (mmol/L)	1.12 ± 0.26	1.05 ± 0.26	0.070

Abbreviations: HF, heart failure; SBP, systolic blood pressure; DBP, diastolic blood pressure; LVEF, left ventricular ejection fraction; CRP, C-reactive protein; NT-pro BNP, N-terminal pro-B-type natriuretic peptide; TnT, troponin T; WBCs, white blood cells; ALT, alanine aminotransferase; GGT, gamma-glutamyltransferase; FBG, fasting blood glucose; Cr, creatinine; TG, triglyceride; TC, total cholesterol; LDL-C, low-density lipoprotein cholesterol; HDL-C, high-density lipoprotein cholesterol.

### 3.2 Baseline Clinical Characteristics According to GGT Tertiles

We further divided all patients into three groups based on the GGT tertiles, and the clinical characteristics of each group were compared. The incidence of HF after myocardial infarction increased significantly with increasing GGT tertiles: The incidence of HF in the first, second, and third GGT tertiles was 7.97%, 14.49%, and 18.38%, respectively (*p* = 0.039). In-hospital mortality also increased with increasing GGT tertiles: The incidence of mortality in each GGT group was 0%, 1.45%, and 5.15%, respectively (*p* = 0.011). Moreover, there were substantial differences in age, CRP levels, white blood cell counts, and ALT, FBG, and creatinine levels between the different GGT tertile groups. More detailed results are shown in Table 2.

**Table 2.** **Patient characteristics according to GGT tertiles**.

	Q1 (n = 138)	Q2 (n = 138)	Q3 (n = 136)	*p*-value
GGT (IU/L)	≤18.9	19–29	>29	
HF (%)	11 (7.97)	20 (14.49)	25 (18.38)	0.039
Female (%)	43 (31.16)	25 (18.12)	19 (13.97)	<0.001
Age (year)	63.17 ± 11.23	58.13 ± 11.95	55.87 ± 14.01	<0.001
Hypertension (%)	57 (41.30)	60 (43.48)	68 (50)	0.322
Diabetes mellitus (%)	23 (16.67)	31 (22.46)	36 (26.47)	0.141
SBP (mmHg)	123.67 ± 18.31	123.13 ± 18.88	126.01 ± 18.24	0.391
DBP (mmHg)	78.49 ± 12.62	79.33 ± 13.74	80.39 ± 12.88	0.484
Anterior myocardial infarction (%)	71 (51.45)	83 (60.14)	73 (53.68)	0.360
Death during hospitalization (%)	0 (0)	2 (1.45)	7 (5.15)	0.011
LVEF (%)	52.35 ± 5.97	52.54 ± 6.18	51.97 ± 6.93	0.762
CRP (mg/L)	3.93 (3.10–8.62)	4.76 (3.10–11.77)	6.45 (3.10–15.00)	0.024
NT-proBNP (pg/mL)	1237 (559.175–1834.5)	818.0 (379.5–1555.0)	744.2 (361.5–2485.5)	0.168
TnT (ng/mL)	4.05 ± 0.79	4.09 ± 0.81	4.08 ± 0.76	0.375
WBCs (×10^9^/L)	9.70 ± 2.66	10.39 ± 3.18	11.45 ± 4.31	<0.001
ALT (IU/L)	33.75 (22.825–49.0)	34.8 (26.0–54.7)	52.85 (34.05–76.45)	<0.001
FBG (mmol/L)	6.87 ± 2.39	7.65 ± 2.92	8.54 ± 3.96	<0.001
Cr (µmol/L)	64.27 ± 15.09	67.98 ± 17.44	71.70 ± 23.64	0.006
TG (mmol/L)	1.24 ± 0.68	1.56 ± 1.06	2.25 ± 1.99	<0.001
TC (mmol/L)	4.55 ± 1.04	4.69 ± 0.99	4.79 ± 1.46	0.255
HDL-C (mmol/L)	1.06 ± 0.25	1.08 ± 0.28	1.03 ± 0.26	0.211
LDL-C (mmol/L)	3.03 ± 0.90	3.11 ± 0.87	3.05 ± 0.99	0.750

Abbreviations: GGT, gamma-glutamyltransferase; HF, heart failure; SBP, systolic blood pressure; DBP, diastolic blood pressure; LVEF, left ventricular ejection fraction; CRP, C-reactive protein; NT-proBNP, N-terminal pro-B-type natriuretic peptide; TnT, troponin T; WBCs, white blood cells; ALT, alanine aminotransferase; FBG, fasting blood glucose; Cr, creatinine; TG, triglyceride; TC, total cholesterol; LDL-C, low-density lipoprotein cholesterol; HDL-C, high-density lipoprotein cholesterol.

### 3.3 Association between GGT Levels and In-hospital HF Risk Complicating STEMI

**Fig. 1.**
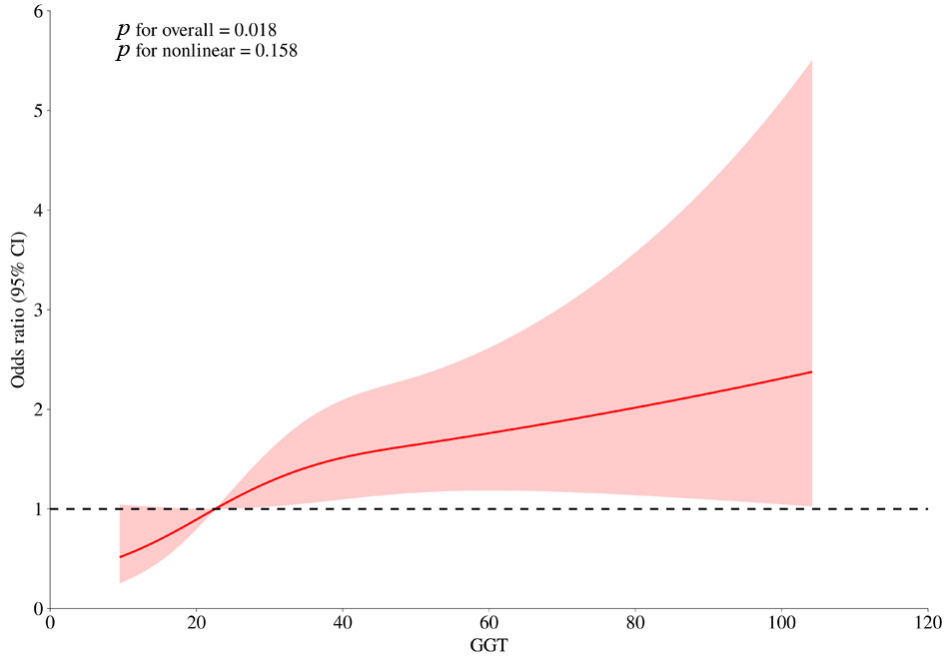
**Restricted cubic splines analysis of GGT with in-hospital HF risk**. GGT, gamma-glutamyltransferase; HF, heart failure.

We used univariate and multivariate logistic regression analyses to evaluate the associations between GGT levels and HF risk after myocardial infarction (Table 3). Univariate logistic regression analysis revealed that the risk of HF in the second and third GGT tertiles was 1.96-fold (95% CI, 0.90–4.26) and 2.60 times (95% CI, 1.22–5.52) greater, respectively, than the first tertile group (*p* for trend = 0.013). After adjusting for sex and age, the HF risk in the second and third GGT tertiles remained significantly greater than the first tertile group, and the ORs were 3.10 (95% CI, 1.35–7.13) and 4.84 (95% CI, 2.12–11.09), respectively (*p* for trend <0.001). According to the multivariate regression analysis, the risk of HF in the second and third GGT tertiles was still significantly greater than in the first GGT tertile after adjusting for several potential covariates (age, sex, hypertension, diabetes, anterior myocardial infarction, and ALT); meanwhile, the ORs were 2.51 (95% CI, 1.06–5.96) and 2.77 (95% CI, 1.13–6.81), respectively (*p* for trend = 0.029). When GGT was used as a continuous variable, each 1-unit increase in lnGGT was related to a 1.88-fold increase in HF risk (odds ratio, OR, 1.88; 95% CI, 1.19–2.96; *p* = 0.007). The RCS suggested a linear relationship between GGT and in-hospital HF (*p* for nonlinearity = 0.158; Fig. 1). These results indicate that elevated GGT levels are an independent risk factor for HF occurrence after myocardial infarction. The results of the univariate analysis of potential covariates (age, sex, hypertension, diabetes, anterior myocardial infarction, and ALT) associated with HF are presented in **Supplementary Table 1**.

**Table 3.** **Odds ratio (95% CI) of HF risk according to GGT tertiles**.

		Q1 (n = 138)	Q2 (n = 138)	Q3 (n = 136)	*p* for trend
GGT (IU/L)		≤18.9	19–29	>29	
HF (%)		11 (7.97)	20 (14.49)	25 (18.38)	0.039
Odd ratio (95% CI)					
	Model 1	1	1.96 (0.90–4.26)	2.60 (1.22–5.52)	0.013
	Model 2	1	3.10 (1.35–7.13)	4.84 (2.12–11.09)	<0.001
	Model 3	1	2.51 (1.06–5.96)	2.77 (1.13–6.81)	0.029
Per 1 lnGGT increment		1.88 (1.19–2.96)			0.007

Model 1: unadjusted OR; Model 2: adjusted for sex and age; Model 3: adjusted for sex, age, hypertension status, diabetes status, anterior myocardial infarction status, and alanine aminotransferase status. GGT, gamma-glutamyltransferase; HF, heart failure; OR, odds ratio.

**Fig. 2.**
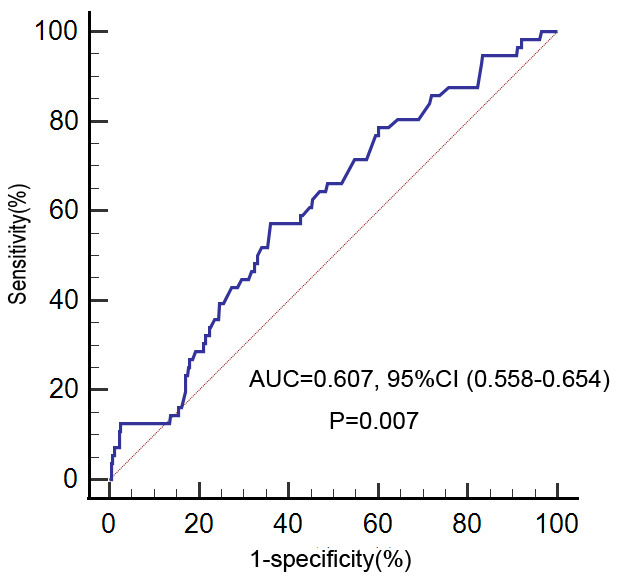
**Receiver operating characteristic curve analysis of GGT with in-hospital HF risk**. GGT, gamma-glutamyltransferase; HF, heart failure; AUC, area under the curve.

The ROC curve analysis was further performed to explore the predictive efficacy of GGT for HF (Fig. 2). The optimal cut-off value of GGT for the prediction of in-hospital HF was 26.6 IU/L, with a sensitivity of 57.14% and a specificity of 64.04% (area under the curve = 0.607; 95% CI, 0.558–0.654; *p* = 0.007).

### 3.4 In-hospital HF and Death Risks

Previous study has shown that HF can significantly increase the risk of early death in patients with myocardial infarction [12]. Therefore, we further analyzed the relationship between HF post-STEMI and in-hospital death. Logistic regression analysis revealed that HF after myocardial infarction significantly increased the risk of in-hospital mortality in STEMI patients after adjusting for sex and age (OR, 7.75; 95% CI, 1.87–32.12; *p* = 0.005).

## 4. Discussion

The mortality rate of acute myocardial infarction has decreased significantly with the development of pharmacological and nonpharmacological treatments. However, the incidence of HF after myocardial infarction remains high; the incidence of in-hospital HF in patients with myocardial infarction is 13–28% [3, 13, 14, 15]. People with HF after myocardial infarction have significantly greater cardiovascular and all-cause mortality [12].

Epidemiological studies have demonstrated a strong association between GGT and cardiovascular diseases [4, 5, 6, 7]. Our study explored the relationship between GGT and HF after primary PCI in STEMI patients. The increase in GGT levels was closely correlated with the incidence of HF in STEMI patients treated with primary PCI: The serum GGT levels in the HF group were significantly greater than those in the non-HF group (28.10 IU/L *vs*. 21.65 IU/L, *p* = 0.001). The incidence of HF increased significantly with increasing GGT tertiles (7.97%, 14.49%, and 18.38%, respectively; *p* = 0.039). A multivariate logistic regression model revealed that the risk of HF in the second and third GGT tertile groups was 2.51 times (95% CI, 1.06–5.96) and 2.77 times (95% CI, 1.13–6.81) greater, respectively, than that in the first GGT tertile group (*p* for trend = 0.029), after adjusting for multiple potential confounding factors. The above results suggest that the elevated GGT levels are an independent risk factor for HF complicating STEMI. The occurrence of HF complicating STEMI significantly increased the risk of in-hospital mortality (OR, 7.75; 95% CI, 1.87–32.12; *p* = 0.005).

The radial approach is routinely used during coronary intervention surgery, and its benefits over the femoral approach have been widely demonstrated [16]. However, patients with cardiogenic shock were often excluded. In our present study, ten patients were diagnosed with cardiogenic shock, and a radial approach was applied to perform these procedures. Accordingly, Tokarek *et al*. [17] also showed that the radial approach was superior to the femoral approach in decreasing periprocedural mortality in STEMI patients with complications following cardiogenic shock. Antithrombotic therapy is an important issue in STEMI patients, especially in patients with atrial fibrillation. Triple antithrombotic therapy or dual antithrombotic therapy remains a vital issue that needs to be carefully considered during clinical practice [18].

The exact pathophysiological mechanism underlying the relationship between GGT levels and HF is still unclear. There are several possibilities: GGT is involved in the metabolism of the antioxidant glutathione and is a sensitive biomarker of oxidative stress [19, 20]. Many reactive oxygen species, such as superoxide anions and hydrogen peroxide, are generated during the redox process of extracellular Fe^3+^ to Fe^2+^ [21]. These reactive oxygen species cause cellular DNA damage, cell proliferation, and apoptosis, promoting HF events after myocardial infarction [22]. Activated GGT has been found to coexist with oxidized low-density lipoprotein (ox-LDL) in foam cells of atherosclerotic plaques and to participate in cardiovascular events by promoting plaque rupturing [23]. Animal experiments have confirmed that GGT activity significantly increases in myocardial tissue after myocardial infarction and can promote myocardial remodeling by downregulating the expression of transient outwards potassium ion current channels [24].

Our study has several limitations and should be cautiously interpreted. First, it was a single-center observational study, and the causal relationship between GGT levels and in-hospital HF could not be analyzed. Second, the sample size was small. Third, we only adjusted for several potential covariates in evaluating GGT and in-hospital HF as possible; however, residual confounding factors may still exist. Lastly, several pieces of surgical information needed to be further analyzed. For example, some procedures performed during the night shift and the operator experience may impact the clinical outcomes.

## 5. Conclusions

In summary, our study demonstrated that elevated GGT levels are positively associated with in-hospital HF and are an independent risk factor for the incidence of in-hospital HF in STEMI patients undergoing primary PCI. Furthermore, people with high GGT levels should be treated with caution during the in-hospital and follow-up periods.

## Availability of Data and Materials

The article’s data will be shared on reasonable request with the corresponding author.

## Supplementary Material

Supplementary material associated with this article can be found, in the online version, at https://doi.org/10.31083/RCM25005.
