# Supplementary material for: Association of Serum Gamma-Glutamyltransferase with In-hospital Heart Failure in Patients with ST-segment Elevation Myocardial Infarction Undergoing Primary Percutaneous Coronary Intervention
Source: Rev Cardiovasc Med. 2025 Jan 8;26(1):25005. doi: 10.31083/RCM25005 (PMC11759973; doi:10.31083/RCM25005)
Supplement: Supplementary file 1 [file 2153-8174-26-1-25005-s1.zip › Supplementary Table 1.docx]

Supplementary Table 1. Univariate analysis.

|  | OR (95%CI) | *p* value |
| --- | --- | --- |
| Age | 1.05 (1.02 ~ 1.07) | <.001 |
| Gender (male) | 0.26 (0.14 ~ 0.47) | <.001 |
| hypertension | 3.00 (1.65 ~ 5.46) | <.001 |
| Diabetes | 2.73 (1.50 ~ 4.97) | <.001 |
| Anterior myocardial infarction | 3.09 (1.61 ~ 5.95) | <.001 |
| Alanine aminotransferase | 1.01 (1.01 ~ 1.02) | <.001 |
