# Supplementary figures and images for: Association of Serum Gamma-Glutamyltransferase with In-hospital Heart Failure in Patients with ST-segment Elevation Myocardial Infarction Undergoing Primary Percutaneous Coronary Intervention
Source: Rev Cardiovasc Med. 2025 Jan 8;26(1):25005. doi: 10.31083/RCM25005 (PMC11759973; doi:10.31083/RCM25005)

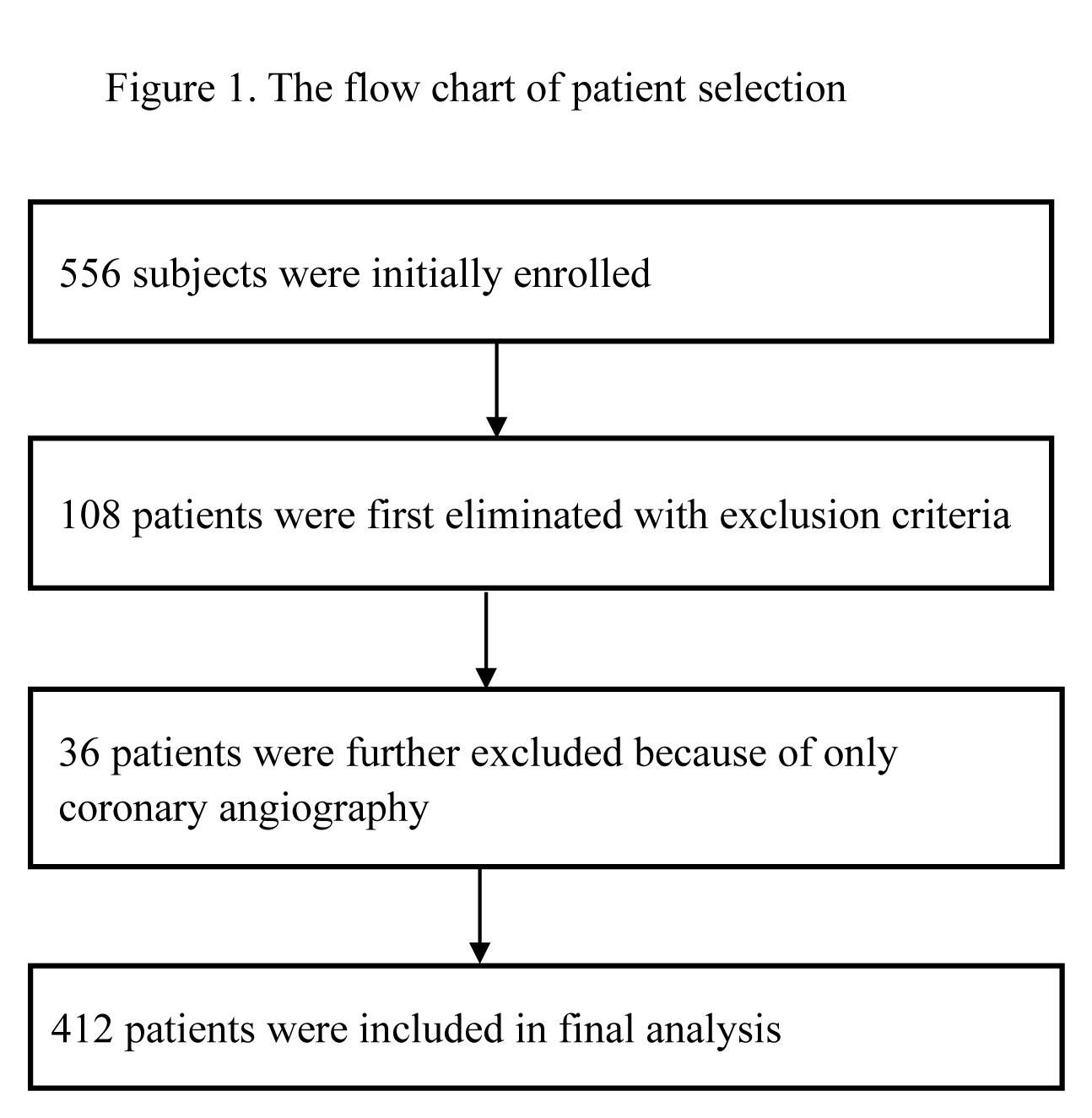

Supplement: Supplementary file 1 [file 2153-8174-26-1-25005-s1.zip › Supplementary Fig. 1.tif]
